# Clinical and Dermoscopic Characteristics of Cutaneous Sarcomas: A Literature Review

**DOI:** 10.3390/diagnostics13101822

**Published:** 2023-05-22

**Authors:** Zoe Apalla, Konstantinos Liopyris, Eirini Kyrmanidou, Christina Fotiadou, Dimitrios Sgouros, Aikaterini Patsatsi, Myrto-Georgia Trakatelli, Evangelia Kalloniati, Aimilios Lallas, Elizabeth Lazaridou

**Affiliations:** 1 Second Dermatology Department, Aristotle University of Thessaloniki, 55535 Thessaloniki, Greece; eirinixx@yahoo.com (E.K.); christinafotiadou@yahoo.com (C.F.); katerinapatsatsi@gmail.com (A.P.); mtrakatelli@hotmail.com (M.-G.T.); evakalloniati@yahoo.gr (E.K.);; 2 First Dermatology Department, National and Kapodistrian University of Athens, 16121 Athens, Greece; konstantinosliopyris@gmail.com; 3 Second Dermatology Department, National and Kapodistrian University of Athens, 16121 Athens, Greece; disgo79@gmail.com; 4 First Dermatology Department, Aristotle University of Thessaloniki, 54124 Thessaloniki, Greece; emlallas@gmail.com

**Keywords:** dermoscopy, cutaneous sarcoma, dermatofibrosarcoma protuberans, atypical fibroxanthoma, Kaposi’s sarcoma, angiosarcoma

## Abstract

Under the umbrella of cutaneous sarcomas (CS) we include a heterogeneous group of rare, malignant, mesenchymal neoplasia, such as dermatofibrosarcoma protuberans, atypical fibroxanthoma, cutaneous undifferentiated pleomorphic sarcoma, cutaneous angiosarcoma and leiomyosarcoma. Clinical presentation and histopathological examination are the cornerstone of CS diagnosis and classification. There are scarce data in the literature in regards to the clinical and dermatoscopic characteristics of CS and the role of dermatoscopy in their early identification. We performed a literature review, aiming to summarize current data on the clinical and dermatoscopic presentation of the most common types of cutaneous sarcomas that may facilitate early diagnosis and prompt management. Based on the available published data, CS are characterized by mostly unspecific dermatoscopic patterns. Dermatofibrosarcoma protuberans, Kaposi’s sarcoma, and in a lesser degree, cutaneous angiosarcoma, may display distinct dermatoscopic features, facilitating their early clinical recognition. In conclusion, dermatoscopy, in conjunction with the overall clinical context, may aid towards suspicion of CS.

## 1. Introduction

Sarcomas are a wide group of solid, malignant neoplasms of mesenchymal origin, including more than seventy histological variants. They are characterized by significant heterogeneity in regards to the age of onset, localization, biologic behavior, disease course and prognosis. The vast majority of sarcomas emerge from the soft tissues, whilst about 20% arise in bones. Based on their incidence that ranges between 4 and 5 per 100,000 per year in Europe, they are considered a rare type of cancer [1,2,3,4]. WHO classification of intermediate and malignant soft tissue neoplasms (Table 1) includes a long list of diverse entities in terms of morphologic, immunophenotypic and biologic behavior characteristics, highlighting the difficulties in accurate recognition and classification of these tumors [5].

Under the term cutaneous sarcomas (CS), we include a plethora of malignant mesenchymal neoplasia that originate either in the dermis or subcutis [1]. The most common entities comprise dermatofibrosarcoma protuberans (DFSP), atypical fibroxanthoma (AFX) and cutaneous undifferentiated pleomorphic sarcoma (CUPS). Kaposi’s sarcoma (KS) and angiosarcoma represent the two major sarcomas of vascular origin involving the dermis and subcutis. Other less common neoplasms are cutaneous liposarcoma and leiomyosarcoma. The majority of CS are characterized by a comparatively good prognosis, as they mostly tend to recur locally, with low rates of distant metastases [1,2,3,4].

CS are far less common compared to other skin malignancies, such as keratinocyte carcinomas and cutaneous melanoma. Delayed diagnosis, with subsequent delays in therapeutic interventions, is a very common scenario in CS [3]. The latter is not only related to their rarity, but can be also attributed to their highly atypical and variable clinical presentation, the lack of public awareness and the poor experience of healthcare professionals with this type of malignancy. Differentiation of CS from other benign and malignant neoplasms is of foremost importance. Clinical presentation and histopathological examination, including immunohistochemistry studies, are the cornerstone of diagnosis of CS [1,2,3,4].

Dermatoscopy (synonyms: dermoscopy, epiluminescence microscopy, incident light microscopy, skin-surface microscopy) is a non-invasive, in vivo diagnostic technique, initially used for the examination of suspicious cutaneous lesions. Dermatoscopy involves the use of a handheld tool, named dermatoscope. The dermatoscopic examination facilitates visualization of anatomic structures that are invisible by naked eye clinical examination. Anatomic alterations visible under the dermatoscope can be located in the epidermis, the dermo-epidermal junction or the papillary dermis. During the process, with the use of specific equipment, physicians can capture dermatoscopic images for storage or for monitoring any kind of changes within a lesion.

There are scarce data in the literature in regards to the dermatoscopic characteristics of CS [2]. The present article aims to summarize current data on clinical and dermatoscopic presentation of the most common types of CS that may facilitate early diagnosis and prompt management.

## 2. Methods

We conducted a web-based search using the following search terms: sarcoma; dermoscopy; dermatoscopy; cutaneous sarcoma; dermatofibrosarcoma protuberans; DFSP; Atypical Fibroxanthoma; AFX; Cutaneous undifferentiated pleomorphic sarcoma; Kaposi Sarcoma; KS; Angiosarcoma; Leiomyosarcoma; Liposarcoma. We identified a total of 96 relevant articles and two of the researchers (Z.A., K.L.) reviewed their content in consensus to decide the literature for inclusion.

## 3. Dermatofibrosarcoma Protuberans (DFSP)

DFSP is considered the most common type of CS. Its rough estimated incidence ranges from 0.8 to 5 cases per million inhabitants, per year. It affects both males and females, with a peak incidence between the second and fifth decade of life. Sites of predilection are the trunk and extremities, but it may ostensibly involve any area of skin [6,7,8,9,10].

DFSP clinically presents as a firm, exophytic plaque with coalescent, brown-red or flesh-colored nodules on its surface. At the initial stage of evolution, DFSP may present as an atrophic, flat plaque, mimicking a scar or scleroderma. Indolent slow growth, often extending for many years is the rule. During childhood, the predominant clinical morphology is that of a flat or slightly elevated lesion, closely mimicking the plaque-type scleroderma. The size of a DFSP lesion is highly variable, depending on time from disease onset, and usually ranges from 2 to 5 cm in largest diameter. Gigantic lesions measuring up to 20 cm have also been reported. Due to the aforementioned characteristics, delayed diagnosis of DFSP is not uncommon [6,7,8,9,10]. Sudden rapid growth may indicate fibrosarcomatous transformation [1,2].

**Figure 1:**
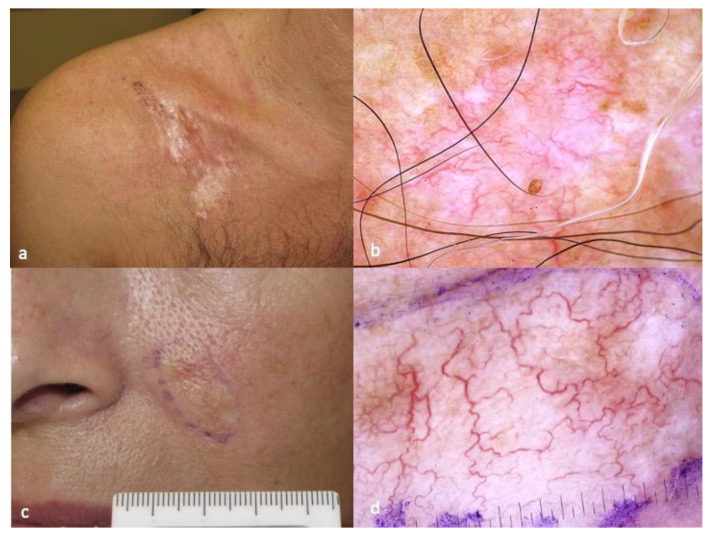
Typical clinical (**a**) and dermatoscopic (**b**) image of DFSP displaying pink-colored background, structureless depigmented areas, structureless light brown areas and linear, arborizing vessels. Morpheic BCC (**c**) closely mimicking DFSP in dermatoscopy (**d**). The photos are from the databases of two of the authors (Z.A., A.L.) and all individuals have provided written informed consent for the use of their photos for scientific purposes.

The dermoscopic pattern of DFSP has been described in case series and case reports, and a fully developed plaque most often consists of a pink-colored background, structureless depigmented areas, structureless light brown areas and vessels that are mostly linear and arborizing (Figure 1) [11,12,13,14,15,16,17,18,19,20]. Shiny white streaks and fine pigment network can be also present. The latter findings are not pathognomonic of DFSP and in certain cases may closely mimic a morpheic BCC (Figure 1) [11,12,13,14,15,16,17,18,19,20].

In terms of histopathology, the tumor is characterized by poorly delimited architecture and presence of spindle cells with a storiform to whorled pattern, largely extending into the subcutaneous tissues. There are monomorphous, minimally pleomorphic, spindle cells with long nuclei and low mitotic activity, intercellular collagen, and small capillaries. The irregular cord-like projections of tumor cells, extending far beyond the center of the tumor, are possibly responsible for the high recurrence rates, even after adequate surgical resection. Amongst the histological variants of DFSP, fibrosarcomatous has the worst prognosis and the pathologist should always report it, if present. Immunohistochemistry for CD34 is particularly useful, since in most of the lesions it is diffusely positive. On the molecular grounds, identification of the t(17;22) (q22;q13) translocation in RT-PCR or FISH test can be highly diagnostic [1,2,8,9,10].

DFSP has a favorable prognosis, with a reported 10-year survival rate reaching 99%. Treatment of choice is Mohs, with complete micrographic control of excisional margins. If Mohs surgery is not available, surgical excision with wide margins is strongly recommended due to high rates of local recurrence. In the scenario of recurrent fibrosarcomatous DFSP, a CT scan of the lungs is recommended, since the lungs represent the most common site of metastasis. Metastatic DFSP may demand systemic treatment with tyrosine kinase inhibitors, such as imatinib. Imatinib can be also used as a second line option for lesions that cannot undergo resection. Considering the high risk of local recurrence, follow-up exams every 6 months for at least 5 years is highly recommended [5,8,9,10].

## 4. Atypical Fibroxanthoma (AFX)

AFX primarily affects elderly individuals and it is mostly seen in chronically sun-exposed areas of the skin, such as the head/neck area. Prevalence and incidence rates of AFX are vastly unknown due to its rarity.

The precise pathogenesis of AFX is still obscure. Mutations in the p53 tumor suppressor gene typically related to a UV signature are often identified [1,2,21,22].

On the clinical basis, AFX presents as a rapidly enlarging, firm, dome-shaped, red or flesh-colored nodule on sun-exposed areas of the skin in elderly individuals. The surface of the lesion is often eroded or crusted. Differential diagnosis includes benign and malignant skin neoplasms, such as NMSCs, DFSP, amelanotic melanoma and pleomorphic dermal sarcoma (PDS) [1,2,21,22,23,24,25,26,27,28].

While some investigators believe there is no distinction between AFX and PDS, others support that they should be classified as different entities. Analytically, AFX is located in the dermis and superficial subcutis, lacking perineural or vascular invasion, as opposed to PDS, which is infiltrating the deeper layers of subcutis, occasionally displaying perineural and/or vascular infiltration. Currently, AFX is considered a neoplasia of intermediate malignancy, based on its limited metastatic potential [21,22,23,24,25,26,27,28].

**Figure 2:**
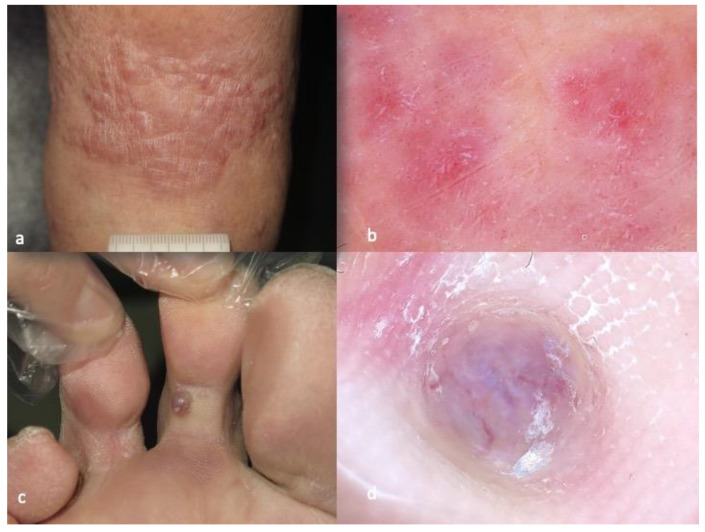
Clinical aspect of patch-type Kaposi’s sarcoma (**a**), displaying purple-pink color in the background, white lines, white clods and rosettes in dermatoscopy (**b**). Clinical aspect of nodular Kaposi’s sarcoma lesion (**c**), characterized by the presence of collaret and polychromatic (“rainbow”) pattern in dermatoscopy (**d**). The photos are from the databases of two of the authors (Z.A., A.L.) and all individuals have provided written informed consent for the use of their photos for scientific purposes.

The dermatoscopic characteristics of AFX are markedly unspecific, simulating other malignant tumors, such as amelanotic melanoma and undifferentiated SCC. As shown in a multicentric study conducted by the International Dermoscopy Society, the majority of AFXs display red and white structureless areas and irregular linear vessels (Figure 2). A pattern with white areas and atypical polymorphous vessels, including linear, dotted, hairpin, arborizing and tortuous vessels in an irregular distribution has also been described [23]. The dermoscopic “rainbow” pattern, a term used to describe areas with various colors mimicking the rainbow on polarized dermoscopy, has been reported in a rare case of aneurysmal AFX [24].

Histopathologic examination and immunohistochemistry are essential for the correct diagnosis. AFX is usually a lesion confined in the dermis and is constantly positive to CD10 and CD68, whilst it is negative to desmin and S-100. The latter immunoprofile is quite unspecific and the definite diagnosis of AFX should be established only after exclusion of other cutaneous spindle cell malignancies, by the use of a wider panel of immunohistochemical stains, such as Sox10 (for melanoma), CK5/6 and/or p63 (for spindle cell SCC), desmin (for tumors originating from the muscles), as well as CD34 or ERG (for angiosarcoma).

Surgical excision is the mainstay of AFX treatment. Recent studies have shown that a median resection margin of 1 cm provides a 98.7% recurrence-free survival and 100% disease-specific survival [22]. Given the extremely low metastatic potential, prognosis of AFX is generally good [21,22].

## 5. Cutaneous Undifferentiated Pleomorphic Sarcoma (CUPS)

CUPS is a rare soft tissue sarcoma with vague etiopathogenesis. Until recently, the tumor was described in the literature as “malignant fibrous histiocytoma”, a term that was abandoned in the last decades [21,25,26,27]. CUPS refers to those CS that cannot be unequivocally classified, despite extensive histological studies. Whether AFX represents the superficial variant of CUPS is still under debate, since both, the histological features and the immunohistochemical profile, literally correspond to those observed in AFX [1,2,20,28,29]. The reason of diagnostic distinction between AFX and CUPS is primarily their different biologic behavior, suggesting a higher likelihood of local recurrence, distant metastasis and tumor-related mortality in CUPS as compared to AFX [28].

The Caucasian population is more commonly affected and the age of onset ranges between 50 and 70 years. About 70% of cases occur among men. In terms of topography, the most affected areas are the thighs and the trunk, whilst head and neck are unusual sites, occurring almost exclusively in adults. CUPS clinically manifests as a gradually enlarging subcutaneous nodule that may ultimately acquire a significant size and ulcerate [1,2,25,26,27,28,29].

**Figure 3:**
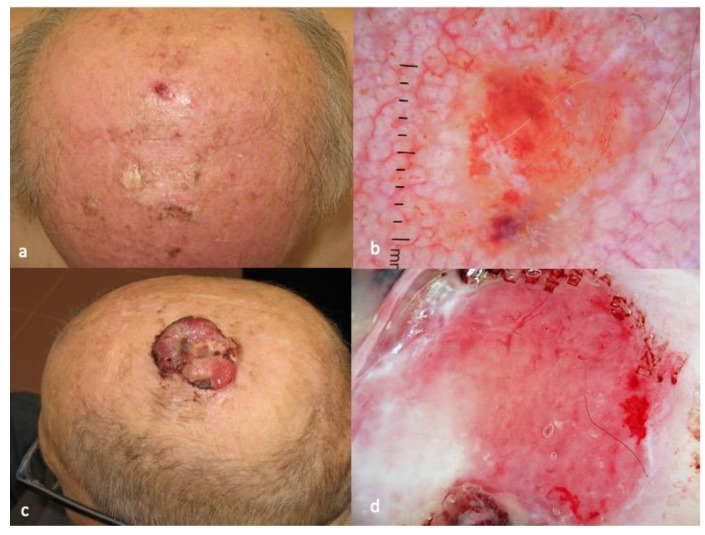
Clinical aspect of AFX (**a**), characterized by red and white structureless areas and irregular linear vessels in dermatoscopy (**b**). Clinical aspect of CUPS (**c**), showing red and white structureless zones in combination with thick, linear irregular vessels in dermatoscopy (**d**). The photos are from the databases of two of the authors (Z.A., A.L.) and all individuals have provided written informed consent for the use of their photos for scientific purposes.

There are scarce data in the literature concerning the dermatoscopic features of CUPS [25,26,27,28,29]. In the few published cases, it was shown that red and white structureless zones were the predominant features, in combination with thick, linear irregular vessels (Figure 3). These clinical and dermoscopic features are definitely non-specific, being shared also by poorly differentiated squamous cell carcinoma.

The gold standard of diagnosis is histological and immunohistochemistry studies that facilitate correct diagnosis and discrimination from other entities with similar morphological alterations [1,2,27,28].

On histological grounds, CUPS is characterized by the presence of pleomorphic histiocyte-like, spindled and multinucleated neoplastic cells, in a fascicular and sheet-like growth. Atypical mitotic activity is almost always present. Differential diagnosis should include other malignant soft tissue neoplasms, such as spindle cell squamous cell carcinoma, angiosarcoma and leiomyosarcoma, as well as desmoplastic melanoma. Immunohistiochemistry is helpful towards differentiation of CUPS from other entities. CUPS is typically positive for CD10, vimentin, CD68 and actin, whilst it is negative for pancytokeratins, CD34, melanoma markers melanA, S100 protein and HMB45 [28]. CUPS is histologically indistinguishable from AFX, which, opposed to CUPS, rarely metastasizes to other organs. Based on pathologists’ recommendations, in the scenario of a tumor >2 cm that extensively invades the subcutis, penetrating the fascia and muscles, or displaying vascular invasion and/or necrosis, the lesion should be diagnosed as CUPS [1,2,28,29].

Wide local excision is the preferential treatment for CUPS due to the high rates of local recurrence, ranging between 19% and 31%. Late-stage diagnosis is related with a poor prognosis, despite the use of adjuvant radiotherapy or chemotherapy [1,2,28,29].

## 6. Kaposi’s Sarcoma (KS)

KS was described in the literature for the first time by a Viennese dermatologist named Moritz Kaposi (1837–1902), about one century ago. Since then, KS has gained much attention among the scientific community, especially after the recognition of its etiologic relation with the acquired immune deficiency syndrome (AIDS). KS is an enigmatic angioproliferative disorder classified into four epidemiological groups, namely AIDS-related (epidemic), iatrogenic (transplant-related), endemic and classic KS. In terms of pathogenesis, all of the aforementioned variants have been linked to the oncogenic virus HHV8 (human herpes virus 8). Classic (or Mediterranean) KS was first described by Kaposi and typically affects middle-aged or elderly individuals. Classic KS has a predilection for males of Mediterranean, East European, or Jewish origin. Endemic KS was originally observed among children and adults in sub-Saharan African populations prior to the arrival of the acquired immunodeficiency syndrome (AIDS). Iatrogenic KS mostly develops in organ-transplant recipients who are receiving chronic immunosuppressive therapy. AIDS-related KS develops in patients living with HIV and represents the most common AIDS-related malignant neoplasm [1,2,30,31,32,33,34].

The clinical presentation of KS is highly heterogenous, with some individuals presenting with latent, indolent disease and others with more aggressive tumors. The skin is the most common site of involvement, followed by mucocutaneous areas, lymph nodes and visceral organs; in rare scenarios, urinary and musculoskeletal systems, heart and eyes, but also endocrine organs may be involved. In regards to the clinical morphology of cutaneous lesions, patch, plaque and nodular type are the most common ones. From the histopathologic point of view, there are numerous additional descriptive variants of KS reported in the literature, such as the hyperkeratotic type, the bullous type, the lymphangioma-like, the ecchymotic type, the telangiectatic, the anaplastic, the keloidal, the pyogenic granuloma-like, the micronodular, the intravascular, the glomeruloid and the pigmented variant of KS. An additional histologic variant of KS, presenting with sarcoid-like granulomas in histology has also been described. The aforementioned descriptive subtypes are rare and not widely accepted [30,31,32,33,34].

**Figure 4:**
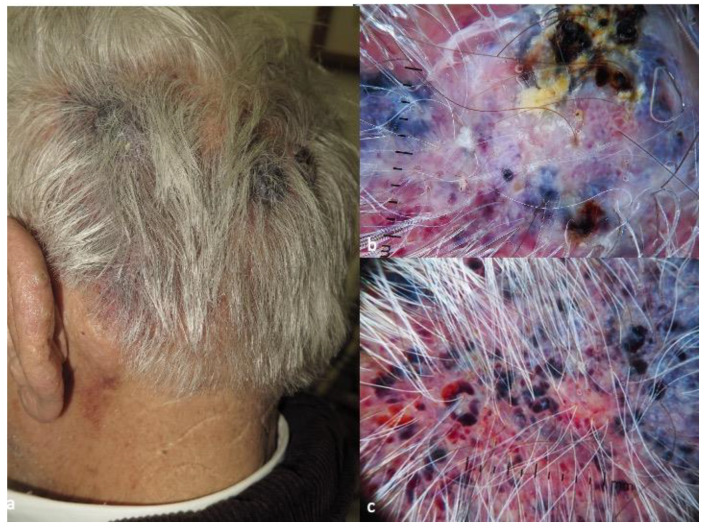
Clinical aspect of CAS (**a**), with dark red, blue and purple dots and clods, divided by white lines and focal hemorrhagic clods in dermatoscopy (**b**). Focally, the dark red clods may mimic the lacunae of hemangioma (**c**). The photos are from the databases of two of the authors (Z.A., A.L.) and all individuals have provided written informed consent for the use of their photos for scientific purposes.

The dermatoscopic pattern of KS is greatly related to the clinical morphology of the lesions [35,36,37,38,39,40]. Analytically, in patches, we mostly observe purple-pink color in the background and white lines, white clods and rosettes (Figure 4). Vessels, present in less than 20% of the cases, are usually dotted and serpentine. Dermatoscopy of plaques is more diverse in terms of vessel morphology and color, with the presence of pink, purple, blue and white. In addition to the white clods and lines, white scales appear in 3 out of 10 KS plaques. Nodular lesions are characterized by the presence of a collaret and polychromatic (“rainbow”) pattern, in about 40% and 50% of cases, correspondingly (Figure 4). Interestingly, both of the latter features are absent in the patch stage. The polychromatic pattern is associated with the vascular lumen-rich histological subtype. On the other hand, rosettes are scarcely seen in KS nodules (~5%). Considering that white lines, “rainbow” pattern and rosettes are seen only under polarized light, contact, polarized dermoscopic examination is highly recommended when suspecting KS [35,36,37,38,39,40].

Histopathologically, KS is characterized by a proliferation of spindle cells, forming cleft-like vascular spaces. A lymphocyte infiltrate admixed with plasma cells is considered characteristic of all variants. The most specific immunohistochemical marker serving towards differentiation of KS from other mimickers is the latency-related nuclear antigen HHV8. While histopathology is literally identical in the different KS clinical subtypes, there are certain differences depending on stage of the disease [30,31,32,33,34].

Analytically, early patch-stage is characterized by atypical vascular spaces, lined by thin endothelial cells, located in the dermis. Promontory sign, referring to ramifying proliferating vessels that commonly surround wider ectatic vessels and skin adnexa, can also be present. Chronic inflammation, foci of hemorrhage and hemosiderin deposition and *hemosiderophages* are common findings in patch-stage KS. Considering that the aforementioned alterations correspond to early histologic changes may be easily missed during histopathologic examination. In this context, clinical information by the physician, raising the suspicion of KS, is of paramount importance for correct diagnosis [30,31,32,33,34].

Plaque-stage and nodular KS display proliferation of both spindle cells and vessels, mostly involving the dermis. The presence of sinuous vascular spaces as a result of the aforementioned proliferation may be sparse in plaque-stage lesions, progressing to formation of fascicles of spindle cells in nodular KS. Intracellular and extracellular hyaline globules can be seen in plaque-stage and nodular-stage KS, while they are extremely rare in patch-stage. Endothelial proliferation is monomorphic with minimal or no atypia in the majority of cases. In the scenario of prominent atypia, the anaplastic variant of KS should be considered [30,31,32,33,34].

With the exception of endemic type, KS is characterized by a good prognosis. Although classic KS rarely requires systemic treatment, systemic therapy may be recommended in symptomatic patients with progressive disease, towards reduction of the size and number of cutaneous lesions and amelioration of disease-related complications and symptoms, including lymphoedema, hemorrhage, pain and functional impairment. Depending on the clinical scenario, treatment options include topical regimens, surgical excision, radiotherapy, highly active antiretroviral therapy, chemotherapy and lately, immunotherapy [1,2,30,31,32,33,34]. Amongst chemotherapeutics, pegylated liposomal doxorubicin and paclitaxel are the most commonly used treatments, independently of the KS clinical variant. There are also reports for vinblastine, gemcitabine, etoposide, bleomycin and vinorelbine in the literature. Recent studies have shown that paclitaxel may also serve as an effective therapy and well-tolerated therapeutic option in individuals suffering from advanced KS [34].

## 7. Angiosarcoma

Angiosarcoma (AS) is a rare and highly aggressive tumor, comprising less than 2% of all soft tissue sarcomas [1,2,40,41,42,43,44,45,46,47,48]. AS originates from lymphatic or vascular endothelial cells and can occur at any age; however, it mainly affects elderly patients. AS comprises a clinically and genetically heterogeneous subgroup of sarcomas and it can occur in any location of the body, with the liver, breast and retroperitoneum being the most common visceral organs affected. However, cutaneous AS (CAS) is the most common presentation (about 60% of cases), particularly of the head and neck [1,2,40,41,42,43,44,45,46,47,48].

CAS represents less than 5% of all skin cancers. It is considered an orphan disease with an annual incidence of about 0.5–1 cases per million population. The etiology of CAS is not well understood; however, some risk factors have been identified. These include exposure to certain chemicals such as arsenic and vinyl chloride, history of radiation therapy and chronic lymphedema. CAS can also occasionally occur as a complication of a pre-existing benign vascular lesion, such as a hemangioma [38,39,40,41,42,46,47,48].

CAS usually presents as a rapidly growing, violaceous or reddish nodule or plaque on the skin, most commonly on the scalp, face, ears and neck. It can also occur on the trunk and extremities. The lesion may have a raised, ulcerated or crusted surface. CAS, at its early presentation, can also mimic benign vascular lesions such as hemangioma or pyogenic granuloma [40,41,42,43,44,45,46,47,48]. Opposed to KS, CAS is an aggressive, rapidly growing tumor.

The diagnosis of CAS is typically made through a combination of clinical examination, dermatoscopy and histopathologic examination. On dermatoscopy, CAS usually presents with dark red and purple or violaceous structureless zones on a light red background. Dark red, blue and purple dots and clods, divided by thick, perpendicular, white lines, reminding the lacuna of hemangiomas, can also be present. Hemorrhagic clods and white and yellow circles, respectively corresponding to focal ulcerations and follicular plugs/opening, are less common. (Figure 2c,d) [43,45].

Upon histology, CAS is seen as an ill-defined infiltrative vascular tumor occupying the dermis and subcutis. In the majority of cases, CAS are moderately differentiated and are characterized by the formation of anastomotic vascular channels of diverse caliber, creating a network-like appearance. As opposed to benign vascular tumors, the vascular channels tend to expand and dissect via the dermal tissue planes. The pathognomonic feature of CAS is the destructive vascular proliferation, dissecting through dermal collagen and engulfing adnexal structures [40,41,42,43,44,45].

The treatment of CAS typically involves surgical excision of the lesion with wide margins (~2 cm if plausible, to decrease the risk for recurrence) followed by radiation therapy and/or chemotherapy. The treatment plan is often tailored to the individual patient and the stage of cancer. Prognosis is generally poor, with relative survival rates for CAS of the head and neck areas ranging between 34% and 39% and 14% and 17% for 5 and 10 years, correspondingly. Most patients with cutaneous angiosarcoma will develop a recurrent or metastatic disease. The management of CAS is challenging due to the rarity of the disease and the lack of standardization of treatment [40,41,42,43,44,45,46,47,48]. The preferred therapeutic intervention is the surgical resection for localized tumors, although extensive infiltration may raise significant challenges in terms of margin control. Recent studies support the need of combined therapeutic modalities, including systemic chemotherapy and irradiation. The latter combination, in addition to surgical excision, have shown to improve the overall survival in a statistically significant way [40,41,42,43,44,45,46,47,48].

## 8. Cutaneous Leiomyosarcoma

Cutaneous leiomyosarcoma (CLMS, synonymous with atypical intradermal smooth muscle neoplasms) is an aggressive, rare type of skin cancer (accounts for less than 1% of all skin cancers) that arises from smooth muscle cells. The majority of CLMS cases occur in individuals over the age of 50 years, with a male-to-female ratio of 2:1. In some cases, a genetic predisposition has been identified in families with multiple cases of CLMS (e.g., TP53 germline mutation carriers) [1,2,49,50,51].

CLMS typically presents as a firm, skin-colored, dermal nodule that may be seen anywhere on the body, but most commonly on the extremities, head/neck region and the trunk. It is typically well-circumscribed and can range in size from a few millimeters to several centimeters. The nodules may be fixed to underlying structures and may occasionally be tender to pressure due to their size and the compression of the surrounding tissues. The overlying skin may be normal or slightly erythematous, with occasional ulceration or crusting [1,2,49,50,51]. Deeper-seated lesions are characterized by worse prognosis. Furthermore, in terms of origin, CLMS is believed to originate from arrector pili muscle cells, whilst subcutaneous lesions arise from muscle cells of the vessels’ wall [1,2,49,50,51].

The diagnosis of CLMS is challenging, as it can mimic a plurality of other skin lesions, including epidermal cysts and skin metastases. The first step in the diagnostic process is a thorough clinical examination, including a detailed history and physical examination, with histopathology being the golden standard for diagnosis. On histopathology, CLMS is characterized by a proliferation of spindle-shaped cells arranged in a fascicular pattern [49,50,51,52].

Dermatoscopy has been shown to be a useful tool in the evaluation of CLMS [52]. The dermatoscopic features of CLMS consist of linear or circular structures with a white to yellow color and homogenous or speckled pigmentation. In addition, dermatoscopy may reveal the presence of telangiectasias and brown to reddish dots or globules [52].

The gold standard in diagnosis remains histopathology and immunohistochemistry studies. CLMS histologically displays high cellularity, with presence of atypical spindle cells arranged in fascicles, bundles, or nodules. Positivity in smooth muscle actin, desmin and h-caldesmon may facilitate discrimination of CLMS from other spindle cell tumors. Description of the depth of infiltration is of paramount importance, considering that dermal localization is linked to a poorer prognosis [1,2,49,50,51]. According to the last WHO classification of soft tissue neoplasms, in the group of malignant smooth muscle tumors, they describe two entities: inflammatory leiomyosarcoma and leiomyosarcoma. Inflammatory leiomyosarcomas are characterized by a less aggressive behavior; however, the data in follow-up of these neoplasms are still very limited [5]. In 2011, Kraft and Fletcher published a large case series of atypical intradermal smooth muscle neoplasms and based on their findings, they concluded that when these neoplasms are restricted to the dermis or appear with only a minimal subcutaneous component, they carry no evident risk of metastasis. In this context, they proposed that the term “atypical intradermal smooth muscle neoplasms” is more appropriate compared to the term “sarcoma” [53]. Currently, this is the most accepted classification in the WHO classification for soft tissue tumors (Table 1).

The treatment of CLMS depends on the size, location and extent of the lesion. Surgical excision with wide surgical margins is usually curative. Both dermal and subcutaneous LMS are characterized by a high rate of recurrence, reported in almost 25% of cases. Considering that CLMS are less responsive to classic chemotherapeutics and radiation therapy, their adjuvant use is still controversial. Opposed to the latter, classic chemotherapy and targeted treatment (mostly tyrosine kinase inhibitors) are recommended for advanced stages. The prognosis for patients with CLMS is generally poor, with a high rate of local recurrence and distant metastasis, especially for deep-seated or subcutaneous lesions [1,2,49,50,51,52].

## 9. Cutaneous Liposarcoma

Cutaneous liposarcoma originates from transformed adipocytes and it is considered an extremely rare malignancy. It mostly affects individuals between 50 and 70 years of age and is clinically characterized by the presence of a subcutaneous nodule, closely resembling an epidermal cyst or a banal lipoma. Histopathology is the only way to establish a definite diagnosis. Upon histopathology, cutaneous liposarcoma can be classified as well-differentiated, dedifferentiated, myxoid, and round cell type. Combinations of the aforementioned subtypes can also be seen [2,54].

As for the majority of cutaneous sarcomas, wide local excision and histolological examination of the margins is the treatment of choice. Adjuvant radiotherapy can be offered in deep infiltrating tumors, whilst chemotherapy is preserved for advanced, metastatic disease [2,54].

Due to their rarity, no dermatoscopic patterns of cutaneous liposarcomas have been described in the literature so far.

## 10. Conclusions

CS represent a heterogenous group of rare tumors characterized by diverse and often unspecific clinical presentations. Due to their rarity and their clinical polymorphea, usually mimicking other malignant but also benign tumors, may pose a significant diagnostic challenge. Early identification, however, is of extreme importance for patient prognosis, especially for those types of CS characterized by aggressive biologic behavior. Herein, we attempted to review the clinical and dermatoscopic features of cutaneous sarcomas. Experience with dermatoscopy indicates that the latter diagnostic technique is useful in raising the suspicion of certain types of CS and could prove a valuable tool towards early recognition. However, due to the scarcity of some of the tumors belonging in the group of CS, there are limited or no data in terms of their dermatoscopic characteristics. In this context, we believe that multicentric studies, facilitating the collection of a larger number of different and rare types of CS are needed, in order to further elucidate the entire spectrum of their clinical and dermatoscopic presentation. In the end, we should also consider that the depth resolution of dermoscopy (and confocal) is around 200 microns. This limit is determined by the scattering of light in the skin. Given that most of these tumors are dermal and often have invasion depth well beyond 200 microns, we cannot expect extensive details on the dermatoscopy features available representing the whole tumor mass.

## Figures and Tables

**Table 1:** Intermediate and malignant soft tissue neoplasms according to the WHO classification.

	Intermediate (Locally Aggressive)	Intermediate (Rarely Metastasizing)	Malignant
**Adipocytic tumours**	Atypical lipomatous tumor		Well-differentiated liposarcoma: lipoma-like, sclerosing, inflammatory Dedifferentiated liposarcoma Myxoid liposarcoma Pleomorphic liposarcoma Myxoid pleomorphic liposarcoma
Fibroblastic/myofibroblastic tumors	Palmar/plantar-type fibromatosis Desmoid-type fibromatosis Lipofibromatosis Giant cell fibroblastoma Dermatofibrosarcoma protuberans	Dermatofibrosarcoma protuberans, fibrosarcomatous Solitary fibrous tumor Inflammatory myofibroblastic tumor Low-grade myofibroblastic sarcoma Superficial CD34-positive fibroblastic tumor Myxoinflammatory fibroblastic sarcoma Infantile fibrosarcoma	Solitary fibrous tumor, malignant Fibrosarcoma NOS Myxofibrosarcoma Low-grade fibromyxoid sarcoma Sclerosing epithelioid fibrosarcoma
So-called fibrohistiocytic tumors		Plexiform fibrohistiocytic tumor Giant cell tumor of soft parts NOS	Malignant tenosynovial giant cell tumor
Vascular tumors	Kaposiform hemangioendothelioma Retiform hemangioendothelioma Papillary intralymphatic angioendothelioma Composite hemangioendothelioma Kaposi sarcoma Pseudomyogenic hemangioendothelioma		Epithelioid hemangioendothelioma Angiosarcoma
Pericytic (perivascular) tumors			Glomus tumor, malignant
Smooth muscle tumors	Smooth muscle tumor of uncertain malignant potential EBV-associated smooth muscle tumor		Inflammatory leiomyosarcoma Leiomyosarcoma
Skeletal muscle tumors			Embryonal rhabdomyosarcoma Alveolar rhabdomyosarcoma Pleomorphic rhabdomyosarcoma Spindle cell/sclerosing rhabdomyosarcoma Ectomesenchymoma
Gastrointestinal stromal tumors			Gastrointestinal stromal tumors
Chondro-osseous tumors			Osteosarcoma, extraskeletal
Peripheral nerve sheath tumors			Malignant peripheral nerve sheath tumor Melanotic malignant nerve sheath tumor Granular cell tumor, malignant Perineurioma, malignant
Tumors of uncertain differentiation	Hemosiderotic fibrolipomatous tumor Angiomyolipoma, epithelioid	Atypical fibroxanthoma Angiomatoid fibrous histiocytoma Ossifying fibromyxoid tumor Myoepithelioma	Phosphaturic mesenchymal tumor, malignant NTRK-rearranged spindle cell neoplasm (emerging) Synovial sarcoma Epithelioid sarcoma: proximal and classic variant Alveolar soft part sarcoma Clear cell sarcoma Extraskeletal myxoid chondrosarcoma Desmoplastic small round cell tumor Rhabdoid tumor Perivascular epithelioid tumor, malignant Intimal sarcoma Ossifying fibromyxoid tumor, malignant Myoepithelial carcinoma Undifferentiated sarcoma Spindle cell sarcoma, undifferentiated Pleomorphic sarcoma, undifferentiated Round cell sarcoma, undifferentiated

## Data Availability

Data are available upon request to the corresponding author.
